# SULT1A1 at a differentiation branch point regulates osteosarcoma cell proliferation and melatonin-mediated anti-tumor activity

**DOI:** 10.3389/fonc.2026.1889414

**Published:** 2026-07-07

**Authors:** Jun Xie, Chao Qian, Jinku Guo, Wei Wang, Chen Chen, Renjie Peng, Ankai Xu

**Affiliations:** 1 Department of Orthopedics, The Quzhou Affiliated Hospital of Wenzhou Medical University, Quzhou People’s Hospital, Quzhou, Zhejiang, China; 2 Department of Nursing, The Quzhou Affiliated Hospital of Wenzhou Medical University, Quzhou People’s Hospital, Quzhou, Zhejiang, China; 3 Department of Orthopedics, Kaihua County Maternal and Child Health Hospital, Quzhou, Zhejiang, China

**Keywords:** Circadian rhythm, differentiation branch point, melatonin, osteosarcoma, SULT1A1

## Abstract

**Background:**

Circadian disruption is increasingly recognized as a contributing factor in tumorigenesis and tumor evolution; however, the role of circadian genes in osteosarcoma cell fate determination and therapeutic response remains to be fully elucidated.

**Methods:**

Gene expression data from GEO were integrated to construct a prognostic model of circadian rhythm-annotated genes based on melatonin-related signatures, validated using the TARGET database. Single-cell RNA-seq and pseudotime trajectory inference were performed to characterize osteosarcoma cell states and identify branch-point genes. We queried publicly available CRISPR-Cas9 dependency data (DepMap) and performed functional assays in osteosarcoma cell lines.

**Results:**

A five-gene circadian rhythm-annotated prognostic signature was established and validated. The intersection of the signature with branch-point genes and circadian rhythm-annotated genes identified *SULT1A1*. CRISPR-Cas9 screening data confirmed that *SULT1A1* is essential for osteosarcoma cell proliferation across multiple cell lines. Experimentally, *SULT1A1* knockdown significantly suppressed cell proliferation and colony formation, and markedly enhanced the anti-tumor efficacy of melatonin in osteosarcoma cells.

**Conclusions:**

Our findings identify *SULT1A1* as a candidate circadian-associated gene localized at an osteosarcoma differentiation branch point, where it may participate in modulating cell proliferation and sensitivity to melatonin. This study uncovers a candidate mechanism potentially linking circadian gene networks to tumor cell state plasticity, with potential implications for understanding non-genetic tumor adaptation and developing chronotherapeutic strategies in osteosarcoma.

## 1 Introduction

Osteosarcoma is an aggressive tumor that primarily occurs in adolescents (1). Although numerous novel therapies are being explored, therapeutic resistance remains a major obstacle. Survival rates are particularly unsatisfactory for patients diagnosed with metastatic disease (2).

Circadian rhythm is an endogenous ~24-hour cycle that regulates physiological processes including sleep-wake cycles, hormone secretion, metabolism, and immune function. Circadian rhythm disruption has been recognized as an important factor in tumorigenesis and tumor progression (3). Circadian rhythm-annotated genes have been used to establish predictive models for clinical outcomes in various cancers, such as lung adenocarcinoma and clear cell renal cell carcinoma (4, 5). However, whether circadian rhythm-annotated genes are involved in the pathogenesis of osteosarcoma and can serve as prognostic biomarkers has not been thoroughly explored. Evidence suggests that melatonin, a key circadian hormone, exerts tumor-suppressive effects in multiple malignancies, yet how its interaction with the circadian gene network influences osteosarcoma cell fate determination remains unclear (4).

In this study, we integrated Gene Expression Omnibus (GEO) microarray data and constructed a circadian gene prognostic model based on melatonin-related features. Using single-cell RNA sequencing, pseudotime trajectory inference, and branch expression analysis modeling (BEAM) analysis, we characterized osteosarcoma cell states and identified *SULT1A1* as a candidate circadian-associated gene located at a differentiation branch point. Functional assays and CRISPR-Cas9 dependency screening confirmed that *SULT1A1* is essential for osteosarcoma cell proliferation and modulates sensitivity to melatonin. In summary, this study uncovers a candidate mechanism linking circadian gene expression to tumor cell state plasticity and provides a theoretical basis for potential chronotherapeutic strategies in osteosarcoma.

## 2 Materials and methods

### 2.1 Data sources and data preprocessing

scRNA−seq data for seven primary osteosarcoma samples (BC2, BC3, BC5, BC6, BC16, BC21, BC22) were obtained from GEO (GSE152048, https://www.ncbi.nlm.nih.gov/geo/). The scRNA-seq data (GSE152048) were processed and analyzed as previously described (6). In brief, quality control, normalization, dimensionality reduction, and clustering were performed using the Seurat package; pseudotime trajectory inference and branch expression analysis modeling (BEAM) were conducted using Monocle2 to identify differentiation branch-point genes. To build a survival cohort, we merged three GEO datasets (GSE21257, GSE16091, GSE39055) with overall survival (OS) data. Batch effects were corrected using ComBat (7). Prognostic samples were also retrieved from Therapeutically Applicable Research to Generate Effective Treatments (TARGET). The TARGET database is an NCI initiative that provides genomic and clinical data specifically for pediatric cancers. From TARGET, we obtained RNA-seq and overall survival data of 95 osteosarcoma patients, which served as an independent external validation cohort. Here, “prognostic samples” refer to patient tumor samples with available overall survival data. Raw FPKM values were converted to TPM, then log2(TPM + 1) transformed and quantile−normalized. After processing, 121 patients (GEO merged) and 95 patients (TARGET) were included. Melatonin−related regulators were sourced from previous studies.

### 2.2 Risk model construction and evaluation

In the GEO cohort, Kaplan−Meier (KM) survival analysis was performed on all genes to identify prognosis−associated genes. The optimal cutoff (ensuring each group ≥30% of patients) was used for grouping, with p < 0.05 considered significant. Circadian rhythm-annotated prognostic genes were identified by Venn diagram and ranked using the randomForestSRC package based on their relative importance scores. To determine the optimal number of top−ranked features, we plotted the out−of−bag (OOB) error rate as a function of the number of top features included. The OOB error rate reached its minimum at five features, after which additional features yielded minimal further improvement (8, 9). Based on this criterion, we selected the top five genes for multivariate Cox regression to construct the final prognostic signature. Time−dependent ROC analysis demonstrated that the CRRSM had moderate predictive performance for 2−, 3−, and 5−year OS, with AUC values of 0.70, 0.66, and 0.64, respectively (**Figure 3B**). Risk score distribution and its association with survival were presented, and a heatmap showed gene expression differences between high− and low−risk groups. Finally, the model was validated using the independent external TARGET database.

### 2.3 Public CRISPR dependency data analysis

We queried the publicly available Cancer Dependency Map (DepMap) database (Broad Institute, https://depmap.org), which contains genome-wide CRISPR-Cas9 knockout screens across hundreds of cancer cell lines. Gene effect scores (CERES) for SULT1A1 were extracted across osteosarcoma cell lines. CERES scores are normalized such that essential genes have negative scores, while non-essential genes have scores near zero; lower (more negative) scores indicate stronger growth dependency on the gene.

### 2.4 Cell lines, drugs, and antibody sources

The human osteosarcoma cell lines HuO9, MG63, and OS152 were obtained from the following sources: HuO9 (YS2745C, 1×10^6^ cells/T25 flask) and MG63 (YS196C, 1×10^6^ cells/T25 flask) from Shanghai Yaji Biotechnology Co., Ltd.; OS152 (CRL-3712) from ATCC. Melatonin was purchased from MedChemExpress (HY-B0075, 5 g). The primary antibodies used were: SULT1A1 polyclonal antibody (Proteintech, 10911-2-AP) and GAPDH monoclonal antibody (Proteintech, 60004-1-Ig).

### 2.5 Gene knockdown assay

Lentiviral shRNA targeting *SULT1A1* and overexpression vectors were constructed by Tsingke Biotechnology. Control vectors included non-targeting shRNA (shNC) and empty vector. The shRNA target sequences were as follows: shNC, 5′-UUCUCCGAACGUGUCACGUTT-3′; sh*SULT1A1* #1, 5′-ACCAAGCGGCTCAAGAATAAA-3′; shSULT1A1 #2, 5′-GAGAAGTTCATGGTCGGAGAA-3′. Lentiviruses were packaged in HEK293T cells using psPAX2/pMD2.G plasmids and Lipofectamine 3000 transfection reagent. Supernatants were collected 48 hours post-transfection, filtered through 0.45 μm membranes, and concentrated. Viral transduction was subsequently performed in HuO9 and OS152 cells with 8 μg/mL polybrene, followed by selection with puromycin (2 μg/mL, 7–10 days). Transduction efficiency was verified by qRT-PCR and Western blot. The 2−ΔΔCT method was used for calculating relative expression values. The primer sequences were as follows: forward, TGTGTGCCCGTCTGTTGTGT; reverse, GAGTCCTGCGTCGAGAGATC.

### 2.6 Western blotting

Cells were washed with ice-cold PBS and lysed in RIPA buffer (Beyotime Biotechnology, China) supplemented with protease and phosphatase inhibitors (Fude Biologic Technology, China). After centrifugation, supernatants were collected for protein quantification and mixed with sample buffer. Proteins were separated by SDS-PAGE and transferred onto PVDF membranes. Membranes were blocked with 5% non-fat milk or BSA, then incubated overnight at 4 °C with primary antibodies against SULT1A1 (1:1000) and GAPDH (1:2000). After washing with TBST, membranes were incubated with HRP-conjugated secondary antibodies. Signals were detected using ECL reagent (Fude Biologic Technology, China). Band intensities were semi-quantified using ImageJ software, and statistical graphs were generated accordingly.

### 2.7 Cell viability and proliferation assays

To evaluate the inhibitory effects of melatonin and SULT1A1 on osteosarcoma cells, the Cell Counting Kit−8 (CCK−8) assay was used. Cells were seeded in 96−well plates, treated under indicated conditions, and incubated for various time intervals. Before measurement, the CCK−8 reagent was diluted 10−fold with complete culture medium. After removing the original medium, 100 μL of the diluted CCK−8 working solution was added to each well. Wells containing PBS served as blanks. The plate was incubated at 37 °C for 2 hours, and the absorbance at 450 nm was measured using a microplate reader. Each condition was tested in at least five replicate wells. Cell viability was calculated using the formula: (Experimental group – Blank group)/(Control group – Blank group). Each condition was tested in three independent biological replicates.

Clonogenic assay was performed to evaluate the inhibitory effect of melatonin and SULT1A1 on osteosarcoma cell proliferation. Cells were seeded at ~2000 cells/well in 6−well plates and cultured for 7 days, with medium changed every two days and 1 mM melatonin administered throughout. After culture, cells were fixed for 1 h, gently washed with PBS, and stained with crystal violet solution (Beyotime Biotechnology, China) for 5–10 min. Following two PBS washes, plates were air−dried, photographed, and colonies were semi−automatically quantified using ImageJ software (version 1.53s). Each condition was tested in three independent biological replicates.

### 2.8 Transwell migration assay

Migration was assessed using Transwell chambers (Corning #3422, 8−µm pore size). Cells were starved for 12 h, then 1×10^5^ cells in 200 µL serum−free medium were added to the upper chamber; the lower chamber contained 600 µL medium with 10% FBS. After 24 h at 37 °C, non−migrated cells were removed. Migrated cells were fixed with 4% paraformaldehyde for 15 min, stained with 0.1% crystal violet for 20 min, and washed three times with water. Five random fields per well were photographed (200×) and counted using ImageJ (Cell Counter). Each condition had three replicates and three independent experiments.

### 2.9 Statistical analysis

Quantitative data from at least three independent experiments are expressed as mean ± standard deviation (SD). Comparisons between two groups were performed using a two−tailed Student’s t−test. For multiple groups, data were compared using one−way or two−way analysis of variance (ANOVA) followed by Dunn’s or Tukey’s *post hoc* test, respectively. All statistical analyses were carried out using R (version 4.0.3) or GraphPad Prism 8 software. Relative protein expression levels in immunoblots were determined using ImageJ software. A p−value < 0.05 was considered statistically significant.

## 3 Results

### 3.1 Screening of circadian rhythm−annotated prognostic candidates

**Figure 1:**
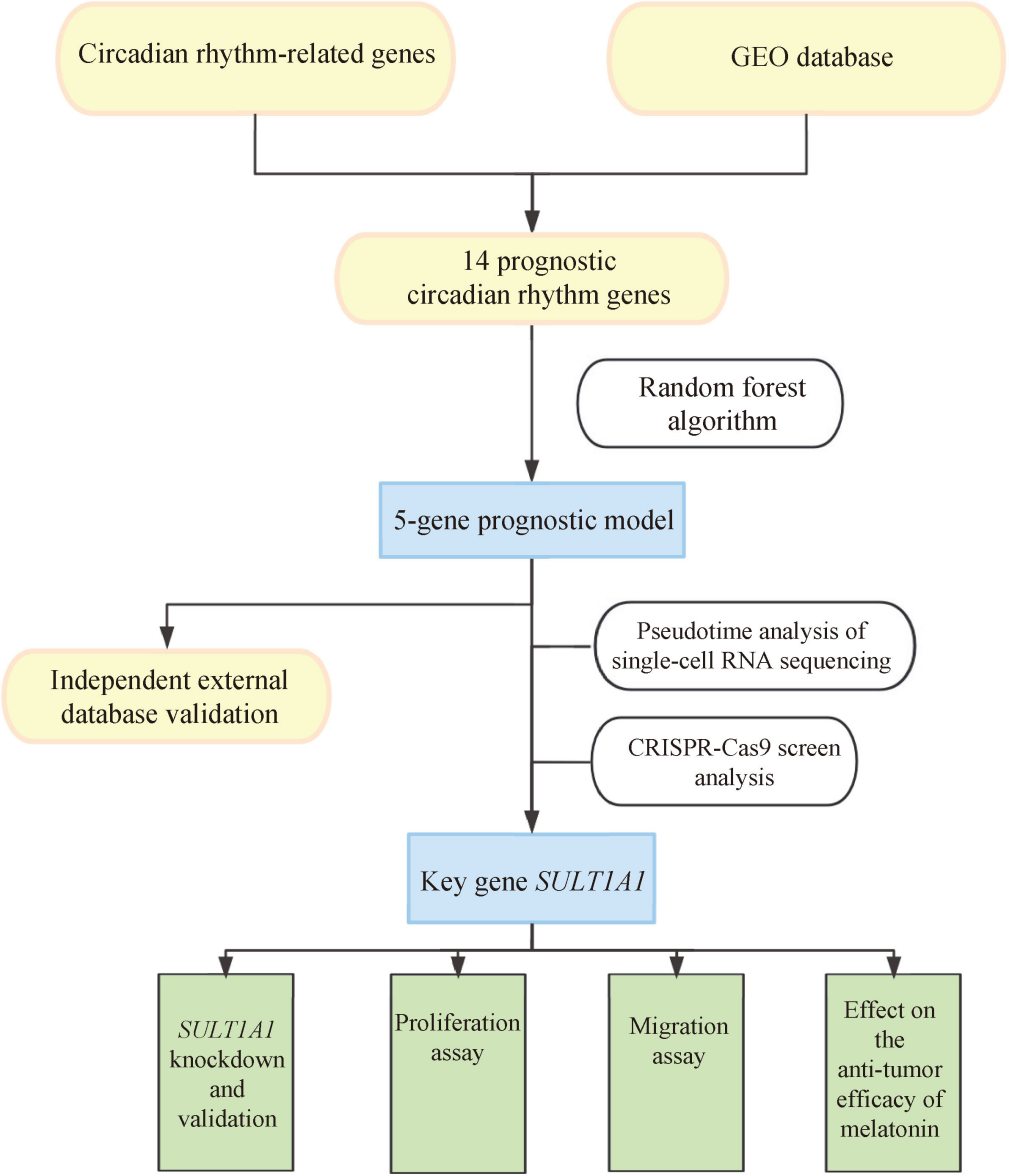
Flowchart of the study design from bioinformatics to experimental validation.

**Figure 2:**
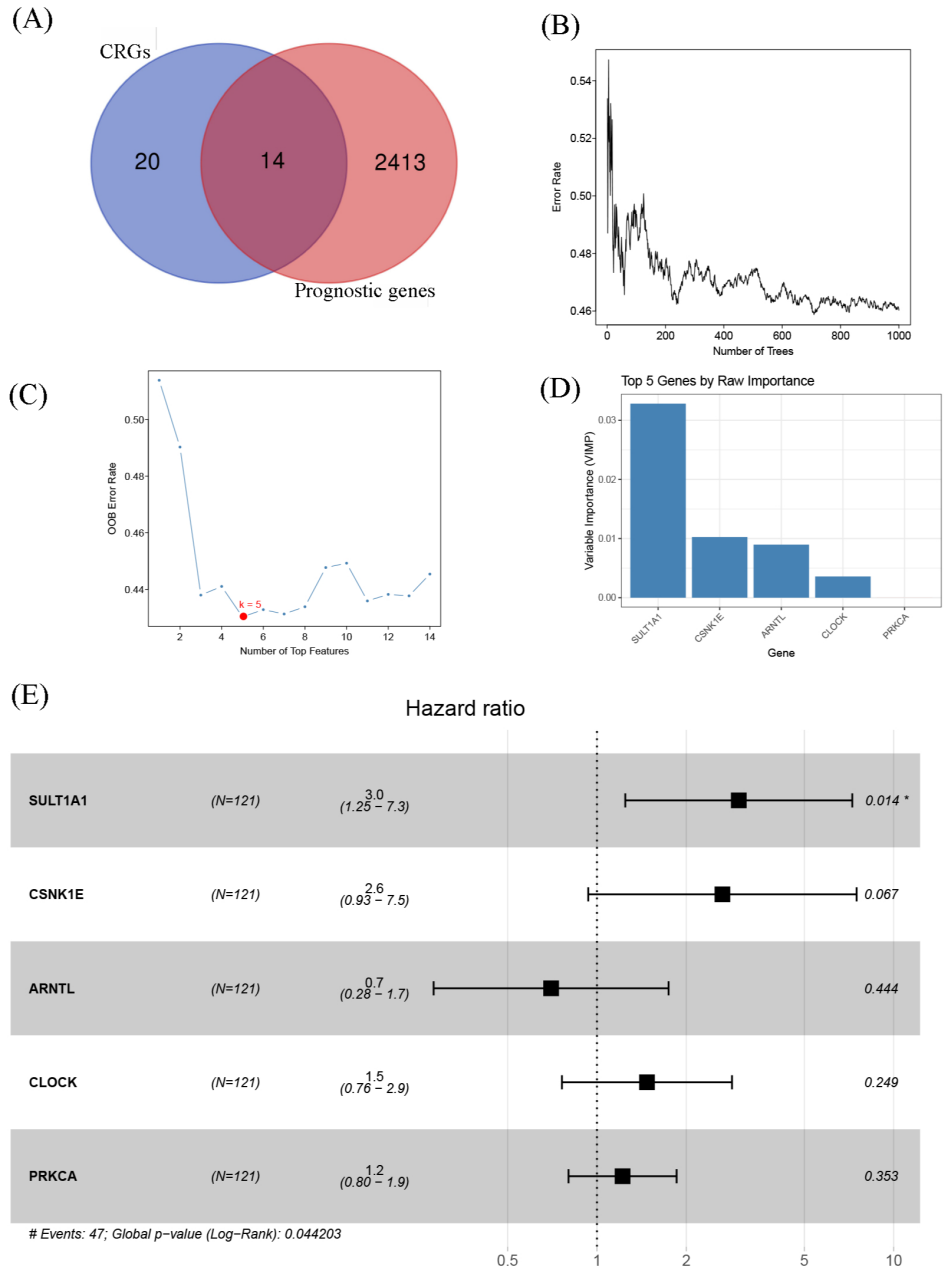
Integration of circadian rhythm-annotated genes (CRGs) with prognostic genes in osteosarcoma and identification of candidate genes via machine learning. **(A)** Venn diagram showing the overlap between CRGs and prognosis−associated genes in osteosarcoma. The intersection represents candidate genes for further analysis. **(B)** Random forest error rate plot. **(C)** Out−of−bag (OOB) error rate as a function of the number of top−ranked features; the minimum was reached at five features. **(D)** Bar chart displaying the variable importance (VIMP) scores for the five most important genes. **(E)** Forest plot of multivariate Cox regression for the five−gene signature. Variables with Hazard ratios (HRs) > 1 and p < 0.05 are considered independent risk factors, while those with HR < 1 are protective factors.

The overall workflow of this study is illustrated in **Figure 1**. To screen for circadian rhythm-annotated genes (CRGs) that are associated with osteosarcoma prognosis, prognostic genes were first identified using KM survival analysis in the GEO osteosarcoma database, and the intersection between CRGs and these prognostic genes was subsequently obtained (5). The Venn diagram revealed a set of overlapping candidate genes (**Figure 2A**). A random forest algorithm was then applied to further screen candidate genes among the overlapping set. The error rate of the random forest model stabilized as the number of decision trees increased (**Figure 2B**). The OOB error rate reached its minimum at five features (**Figure 2C**), and the top five genes with the highest relative importance — *SULT1A1, CSNK1E, ARNTL, CLOCK*, and *PRKCA* — were selected (**Figure 2D**). Multivariate Cox regression analysis demonstrated that, among these genes, *SULT1A1* was independently associated with OS (p < 0.05), as shown in the forest plot (**Figure 2E**). These results indicate that specific CRGs may serve as potential prognostic markers in osteosarcoma.

### 3.2 Prognostic risk score generation and validation

**Figure 3:**
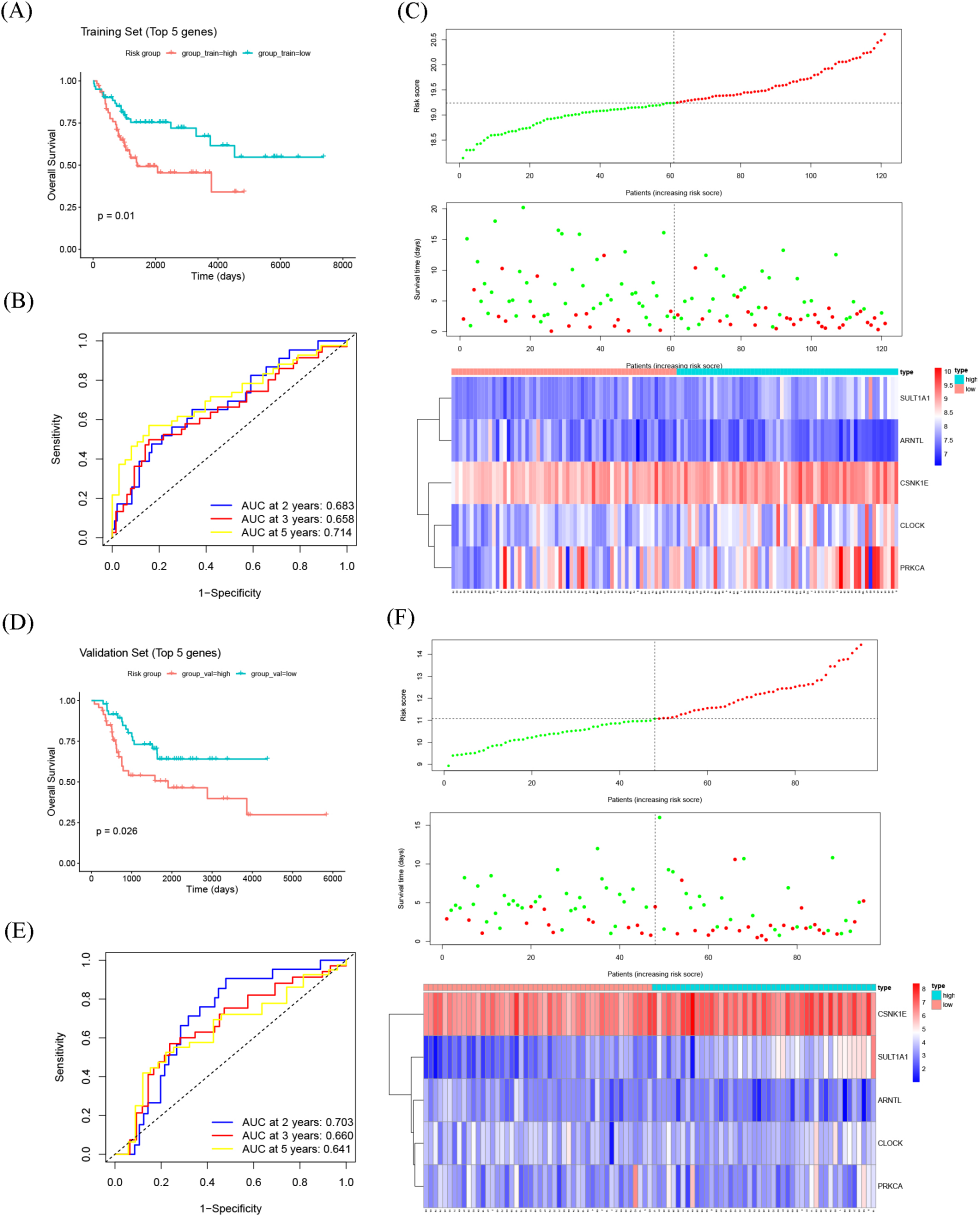
Validation of the circadian rhythm-annotated gene−based risk score model (CRRSM) in osteosarcoma patients. **(A)** Kaplan−Meier survival curve showing the overall survival (OS) of high−risk and low−risk patients in the GEO cohort. The log−rank test was used to compare survival differences between the two groups. **(B)** Time−dependent receiver operating characteristic (ROC) curve analysis evaluating the prognostic performance of CRRSM for predicting 2−, 3−, and 5−year OS in the GEO cohort. The area under the curve (AUC) values are indicated in the figure. **(C)** Risk score distribution, patient survival status, and expression profiles of the circadian rhythm-annotated prognostic genes in the GEO cohort. Patients were divided into high−risk and low−risk groups based on the median risk score. **(D–F)** Similar analyses performed in the TARGET cohort using the same median cutoff value.

A circadian rhythm−annotated gene−based risk score model (CRRSM) was constructed using the identified genes. In the GEO cohort, KM survival analysis showed that patients in the high-risk group had significantly worse OS compared with those in the low-risk group (**Figure 3A**). Time dependent ROC analysis demonstrated that the CRRSM had moderate predictive performance for 2-, 3-, and 5-year OS, with AUC values as indicated (**Figure 3B**). The risk score distribution, patient survival status, and expression profiles of the model genes in the GEO cohort are shown in **Figure 3C**. Similar results were obtained in the independent TARGET cohort, where high risk patients also exhibited poorer OS (**Figures 3D–F**). These findings confirm the prognostic value of the CRRSM in osteosarcoma.

### 3.3 SULT1A1 identified as a candidate gene

**Figure 4:**
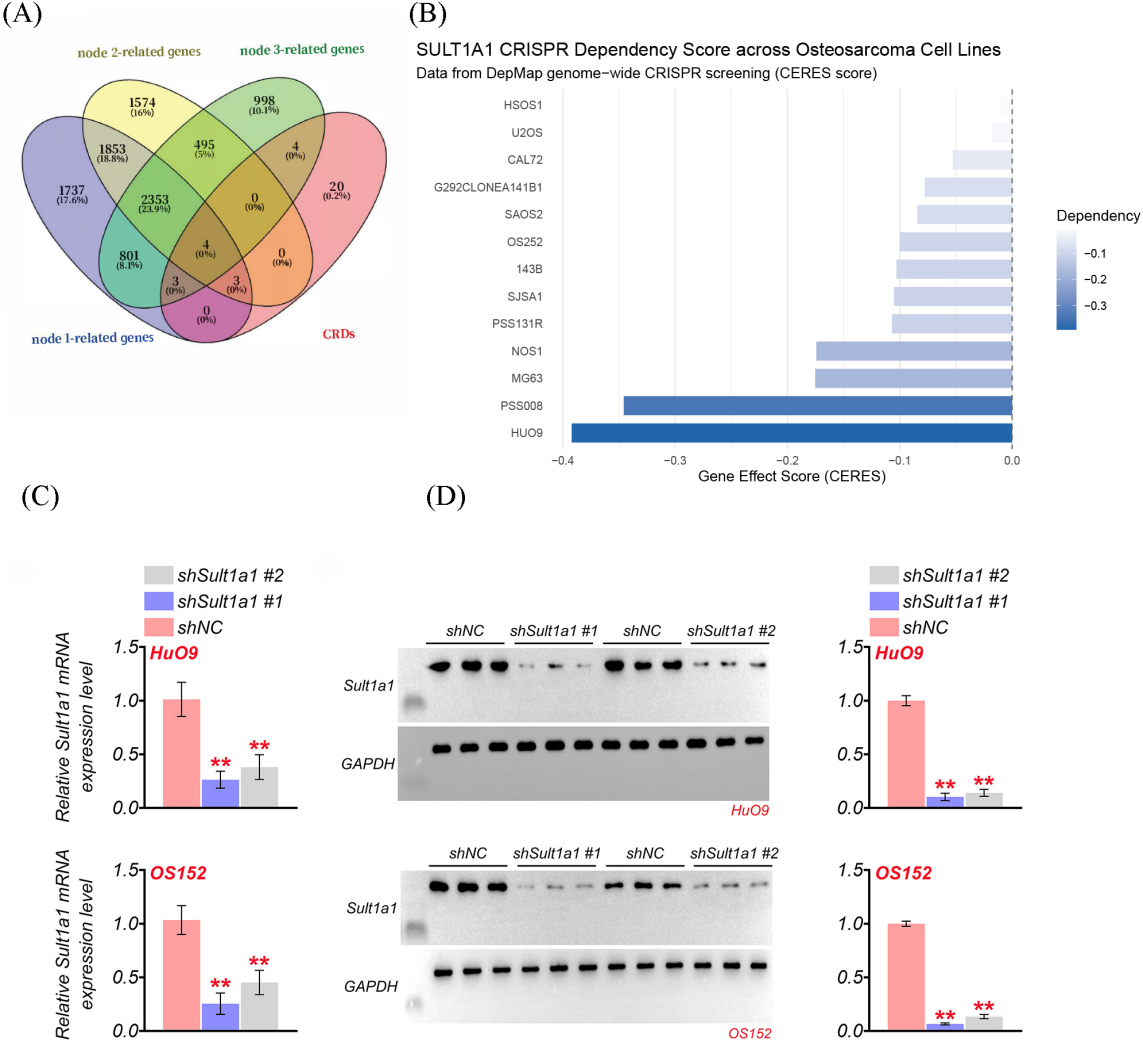
Identification of SULT1A1 as a candidate gene integrating circadian rhythm-annotated gene sets and single−cell differentiation trajectories, and its dependency in osteosarcoma cells. **(A)** Venn diagram showing the overlap between circadian rhythm-annotated genes (CRGs) and genes associated with differentiation branch points identified by single−cell RNA−seq pseudotime analysis. The intersection represents candidate genes potentially linking circadian-annotated gene networks to osteosarcoma differentiation. **(B)** Genome−wide CRISPR screening results from the DepMap database. The bar plot shows the gene effect scores (CERES) upon knockout of SULT1A1 across different osteosarcoma cell lines. Lower (more negative) CERES scores indicate stronger growth dependency on the gene. **(C)** Validation of SULT1A1 knockout efficiency by quantitative PCR (qPCR). **p < 0.01 compared with the control group. Data are presented as mean ± SD (n = 3 independent experiments). **(D)** Western blot analysis of SULT1A1 protein expression after SULT1A1 knockout (left panel) and the corresponding densitometric quantification normalized to a loading control (right panel). **p < 0.01. Data are representative of three independent experiments.

To identify a candidate gene potentially linking circadian-annotated gene networks to osteosarcoma progression, we overlapped CRGs with differentiation branch point related genes derived from single cell RNA seq pseudotime analysis. The Venn diagram revealed four overlapping genes: *CALM1, CYP1B1, SULT1A1*, and *PER3* (**Figure 4A**). Among these, we further evaluated their potential dependency in osteosarcoma using publicly available genome−wide CRISPR screening data from the DepMap database. As shown in **Figure 4B**, according to the DepMap data, knockout of *SULT1A1* resulted in negative gene effect scores (CERES) across multiple osteosarcoma cell lines, indicating that osteosarcoma cells are highly dependent on *SULT1A1* for growth. Therefore, *SULT1A1* was selected for subsequent validation. qPCR confirmed that SULT1A1 mRNA expression was significantly reduced in shSULT1A1−transduced cells compared with shNC controls (p < 0.01; **Figure 4C**). Western blot analysis and densitometric quantification further confirmed a marked decrease in SULT1A1 protein expression upon knockout (p < 0.01; **Figure 4D**). Together, these data identify *SULT1A1* as a candidate circadian-associated gene that warrants further investigation in osteosarcoma.

### 3.4 *SULT1A1* knockdown inhibits osteosarcoma growth/migration

**Figure 5:**
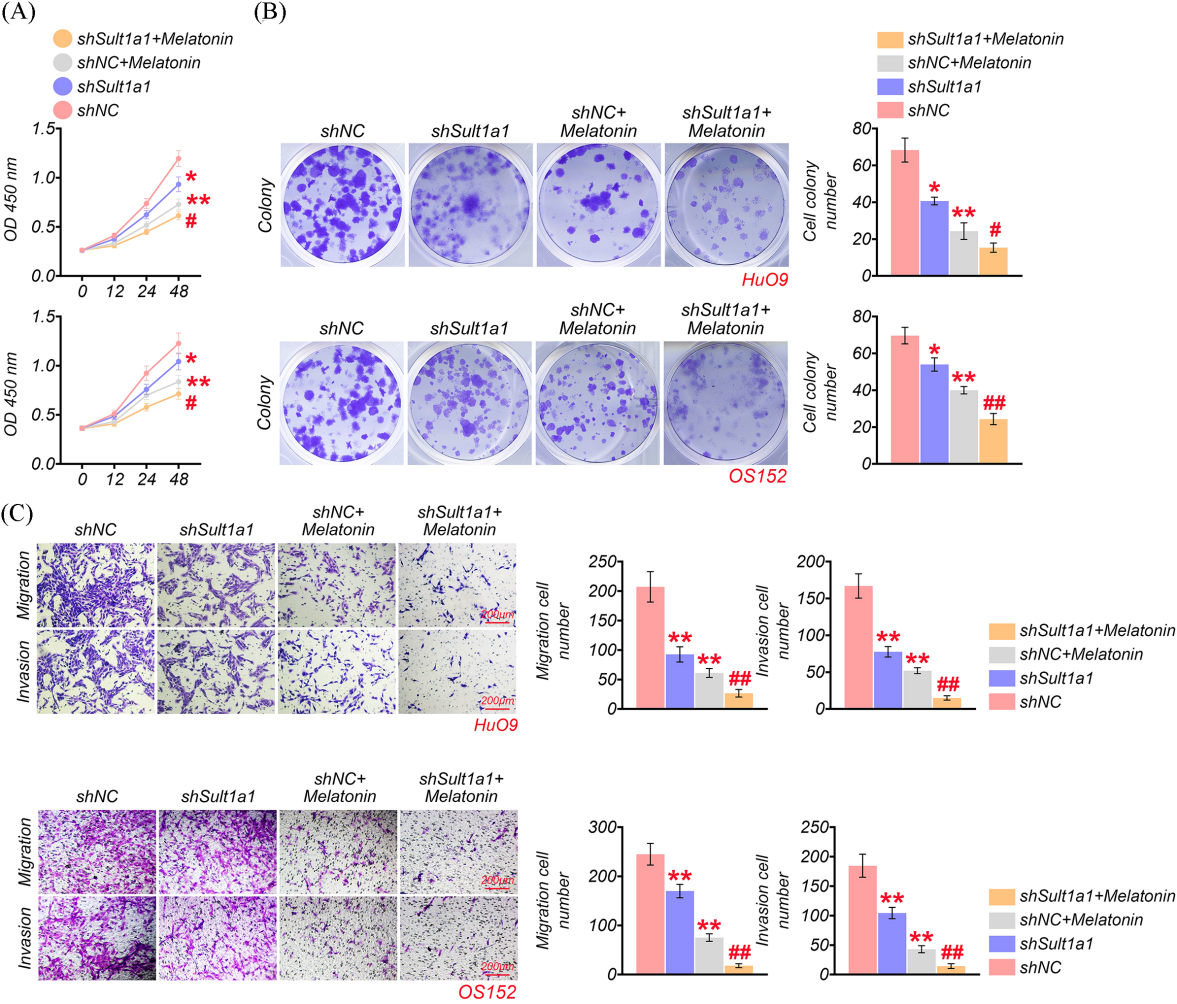
Suppression of SULT1A1 enhances the anti−proliferative and anti−migratory effects of melatonin in osteosarcoma cells. **(A)** Time−dependent cell viability (OD450) curves of HuO9 and OS152 cells under four conditions: shNC (control), shSULT1A1, shNC + melatonin, and shSULT1A1 + melatonin. Data are presented as mean ± SD (n = 3). *p < 0.05, **p < 0.01 (*vs*. shNC); #p < 0.05, ##p < 0.01 (*vs*. shNC + melatonin). (Two−way ANOVA with appropriate *post hoc* test). **(B)** Representative images of colony formation assays (left panel) and quantitative analysis of colony numbers (right panel) for HuO9 and OS152 cells under the four treatment conditions. Data are expressed as mean ± SD (n = 3). *p < 0.05, **p < 0.01 (*vs*. shNC); #p < 0.05, ##p < 0.01 (*vs*. shNC + melatonin). **(C)** Representative images of Matrigel−based transwell migration assays (left panel) and quantification of migrated cells per field (right panel) for HuO9 and OS152 cells under the same four conditions. Scale bar = 200 μm. Data are shown as mean ± SD (n = 3). *p < 0.05, **p < 0.01 (*vs*. shNC); #p < 0.05, ##p < 0.01 (*vs*. shNC + melatonin).

We first evaluated the effect of melatonin on osteosarcoma cell viability. As shown in **Supplementary Figure**, melatonin treatment reduced OD450 values in HuO9, MG63 and OS152 cells in a dose and time dependent manner, with significant inhibition at higher concentrations and longer treatment times. To determine the functional role of SULT1A1, we performed loss of function experiments in HuO9 and OS152 cells. Compared with shNC controls, sh*SULT1A1* cells showed significantly reduced cell proliferation over time (**Figure 5A**). Colony formation assays revealed that *SULT1A1* knockdown alone markedly decreased colony number (**Figure 5B**). Matrigel transwell migration assays demonstrated that *SULT1A1* knockdown also suppressed cell migration (**Figure 5C**). These results indicate that *SULT1A1* knockdown inhibits osteosarcoma cell growth and migration.

### 3.5 SULT1A1 knockdown boosts melatonin’s anti−tumor effect

We next investigated whether *SULT1A1* knockdown enhances the anti-tumor effect of melatonin. HuO9 and OS152 cells were treated under four conditions: shNC, sh*SULT1A1*, shNC+melatonin, and sh*SULT1A1*+melatonin. As shown in **Figure 5A**, melatonin treatment alone significantly reduced cell viability, and the combination of *SULT1A1* knockdown with melatonin resulted in the strongest inhibition, with significant differences compared with shNC+melatonin. Colony formation assays (**Figure 5B**) demonstrated that while either melatonin or *SULT1A1* knockdown alone reduced colony number, their combination further suppressed colony formation, as indicated by the comparison between sh*SULT1A1*+melatonin and shNC+melatonin. Matrigel transwell migration assays (**Figure 5C**) revealed that the combination treatment also led to the most pronounced decrease in cell migration. Collectively, these data indicate that *SULT1A1* knockdown boosts the anti-proliferative and anti-migratory effects of melatonin in osteosarcoma cells.

## 4 Discussion

Circadian rhythm disruption has been increasingly linked to tumorigenesis, tumor progression, and treatment resistance through mechanisms including cell cycle dysregulation, DNA damage repair impairment, metabolic reprogramming, and immune surveillance modulation (10). Core circadian regulators such as *BMAL1, CLOCK, PER*, and *CRY* interact with multiple oncogenic pathways, including p53, MYC and PI3K/AKT, to shape tumor behavior (10–12). Moreover, core molecular clock factors have recently been shown to be critical for osteosarcoma stem cell survival and behavior via direct modulation of cancer stem cells and lipid metabolic pathways (12). Despite these advances, the role of circadian rhythm-annotated genes in osteosarcoma remains incompletely characterized, highlighting an important gap in current understanding.

We identified five circadian rhythm-annotated genes—*ARNTL (BMAL1), CSNK1E, CLOCK, PRKCA* and *SULT1A1*—as prognostic markers in osteosarcoma. ARNTL, also known as BMAL1, encodes a core circadian transcription factor that, together with CLOCK, drives rhythmic expression of thousands of genes. *ARNTL* expression is aberrantly altered in multiple cancers, and its dysregulation has been associated with tumor burden and poor survival in lung adenocarcinoma models (11, 13). However, consistent with previous studies, ARNTL was a protective factor for osteosarcoma patient prognosis, suggesting that avoiding circadian rhythm disruption may confer a protective effect in osteosarcoma patients (14). *CSNK1E* encodes casein kinase 1 epsilon, a key regulator of the circadian clock that phosphorylates and degrades PER proteins to maintain the ~24-hour rhythm. In hepatocellular carcinoma, CSNK1E was identified as a robust independent prognostic biomarker, and CSNK1E knockdown significantly suppressed cell proliferation, migration, and invasion while promoting apoptosis (15). Moreover, multi-omics and single-cell sequencing analyses revealed that CSNK1E is significantly up-regulated in most tumors and that circadian pathway scores are significantly associated with OS in cancer patients (13). SULT1A1, the third component of our prognostic model, belongs to the cytosolic sulfotransferase family and is primarily involved in the sulfonation and metabolic clearance of various endogenous and exogenous compounds, including hormones and drugs. Importantly, SULT1A1 has been detected in human osteosarcoma cell lines, with characteristics similar to those expressed in other human tissues (16). CLOCK, the core transcriptional activator of the circadian feedback loop, exerts non−canonical oncogenic functions beyond rhythm regulation: it promotes glioblastoma angiogenesis via the OLFML3−POSTN−TBK1 axis, and collaborates with BMAL1 to enhance homologous recombination repair of DNA double−strand breaks, thereby driving chemoresistance (17, 18). Meanwhile, PRKCA (PKCα) participates in light−induced clock resetting by modulating BMAL1 phosphorylation and PER2 stability, and exhibits context−dependent roles in cancer—driving proliferation via MAPK/ERK and PI3K/AKT in most tumors, yet potentially suppressing prostate cancer through ferroptosis (19, 20). The prognostic model combining these five genes was validated in independent GEO and TARGET cohorts, with high-risk patients showing significantly worse OS. Previous studies have constructed lncRNA-based circadian rhythm-related prognostic signatures in osteosarcoma (21). To our knowledge, this is the first protein-coding gene-based circadian rhythm-annotated prognostic model specifically established in osteosarcoma.

In recent years, single-cell RNA sequencing has revolutionized the understanding of tumor heterogeneity by enabling transcriptome profiling at the single-cell level (22). Pseudotime analysis, a computational approach that reconstructs continuous biological trajectories from single-cell gene expression data, has been extensively applied to infer developmental lineages and identify genes associated with differentiation branch points (23). Specifically, BEAM is a widely used algorithm for identifying genes that exhibit branching expression patterns during cell fate decisions, comparing two models with a likelihood ratio test for branch-dependent expression. Complementing these transcriptomic approaches, the Cancer Dependency Map (DepMap) project—a large-scale public-private consortium led by the Broad Institute—has systematically performed genome-wide CRISPR-Cas9 knockout screens across hundreds of cancer cell lines to catalog gene dependencies and cancer vulnerabilities (24). The DepMap CRISPR knockout pipeline generates large-scale high-quality screening data for identifying genetic dependencies across a wide range of human cancer cell lines. Leveraging these powerful technologies, we intersected circadian rhythm-annotated genes with differentiation branch point-related genes identified through pseudotime and BEAM analysis of osteosarcoma single-cell transcriptomic data. The Venn diagram revealed four overlapping candidates—*CALM1, CYP1B1, SULT1A1*, and *PER3*—among which DepMap CRISPR screening data further demonstrated that SULT1A1 knockout produced negative CERES scores across multiple osteosarcoma cell lines, indicating high growth dependency. Thus, *SULT1A1* emerged as the most promising candidate gene potentially linking circadian-annotated gene networks to osteosarcoma cell fate determination and tumor progression.

*SULT1A1* encodes a thermostable phenol sulfotransferase that catalyzes the sulfate conjugation—and thereby the metabolic inactivation—of a wide range of substrates, including thyroid hormones, estrogens, neurotransmitters, drugs, and xenobiotics (25). The enzyme is expressed in various human tissues. Importantly, SULT1A1 has been detected in human osteosarcoma and mature osteoblast cell lines, with biochemical characteristics similar to those expressed in other human tissues (16). SULTs may exert regulatory roles in the deactivation of thyroid hormones and estrogenic compounds in bone, potentially affecting hormone action and skeletal responses. Moreover, SULT1A1 has been implicated in cancer risk modulation through the metabolic activation of carcinogenic compounds. In our wet experiments, SULT1A1 knockout significantly suppressed osteosarcoma cell proliferation, colony formation, and migration (**Figures 5A–C**). Notably, SULT1A1 knockdown markedly enhanced the anti-tumor effect of melatonin, an endogenous circadian hormone. Melatonin has emerged as a promising adjunctive agent in oncology due to its pleiotropic biological actions, including redox regulation, anti-inflammatory and oncostatic effects (26). Mechanistically, it has been demonstrated that SULT1A1 plays a key role in the biotransformation of melatonin. The active melatonin metabolite, 6-hydroxymelatonin (6-OHMel), is conjugated by SULT1A1 to form 6-hydroxymelatonin sulfate (6-OHMelS). Overexpression of SULT1A1 in breast cancer cells resulted in dramatic increases (up to 110-fold) in 6-OHMelS formation in cellular supernatants (27). Thus, SULT1A1 knockdown likely reduces melatonin metabolic clearance, leading to sustained local drug accumulation and prolonged anti-tumor activity. Given that SULT1A1 knockout and melatonin treatment produced the strongest inhibitory effects across all functional assays, our data suggest that targeting SULT1A1 may represent a novel strategy to sensitize osteosarcoma cells to melatonin-based interventions. Collectively, these findings identify *SULT1A1* as a candidate circadian-associated gene that may influence osteosarcoma cell viability and melatonin sensitivity. These results establish a rationale for further investigation of SULT1A1-targeted combination therapy with melatonin, although direct evidence of SULT1A1 rhythmic expression and clock regulation in osteosarcoma cells remains to be established.

## 5 Limitations

Several limitations should be noted. First, our prognostic model was built on public datasets with limited sample sizes, and its moderate AUC (0.64–0.71) requires validation in larger prospective cohorts. Second, functional experiments were confined to two cell lines without *in vivo* validation. Third, although *SULT1A1* was identified from circadian-annotated gene sets and is reportedly under circadian control in other tissues, we did not measure its rhythmic expression in osteosarcoma cells, nor whether its knockdown affects the core clock. Future studies using U2OS Per2:Luc cells could address this (28). Therefore, our findings identify *SULT1A1* as a candidate circadian-associated gene that may influence osteosarcoma cell behavior, rather than establishing a mechanistic link to circadian clock function. Future validation in patient-derived xenograft models and quantification of melatonin metabolites would also be valuable.

## 6 Conclusion

In conclusion, this study establishes a five−gene prognostic model (CRRSM) for osteosarcoma and identifies *SULT1A1* as a candidate circadian-associated gene that may influence tumor cell proliferation and melatonin sensitivity. Our findings provide a rationale for further investigation of SULT1A1-targeted combination therapy with melatonin, although direct evidence of its circadian regulatory role in osteosarcoma remains to be established.

## Data Availability

The datasets presented in this study can be found in online repositories. The names of the repository/repositories and accession number(s) can be found below: https://www.ncbi.nlm.nih.gov/geo/ (GSE152048, GSE21257, GSE16091, GSE39055) and https://www.cancer.gov/ccg/research/genomesequencing/target (Therapeutically Applicable Research to Generate Effective Treatments).

## Supplementary material

Supplementary Figure 1 Effects of melatonin on cell viability and proliferation inhibition in osteosarcoma cell lines. **(A)** Line graphs showing OD450 values of (top) HuO9, (middle) MG63, and (bottom) OS152 cell lines treated with increasing concentrations of melatonin at different time points. Data are presented as mean ± SD from three independent experiments. Asterisks indicate significant differences compared with the control group (0 mM melatonin) at the corresponding time point (*p < 0.05, **p < 0.01; two−way ANOVA followed by *post hoc* test). **(B)** Inhibition rates of HuO9, MG63, and OS152 cells after treatment with various concentrations of melatonin for 24 h and 48 h. The inhibition rate was calculated as [1 – (OD450 of treated group/OD450 of control group)] × 100%. Data are expressed as mean ± SD (n = 3). Significant differences relative to the control are marked with asterisks (*p < 0.05, **p < 0.01; multiple t−test or two−way ANOVA).
