# Supplementary figures and images for: SULT1A1 at a differentiation branch point regulates osteosarcoma cell proliferation and melatonin-mediated anti-tumor activity
Source: Front Oncol. 2026 Jul 7;16:1889414. doi: 10.3389/fonc.2026.1889414 (PMC13386055; doi:10.3389/fonc.2026.1889414)

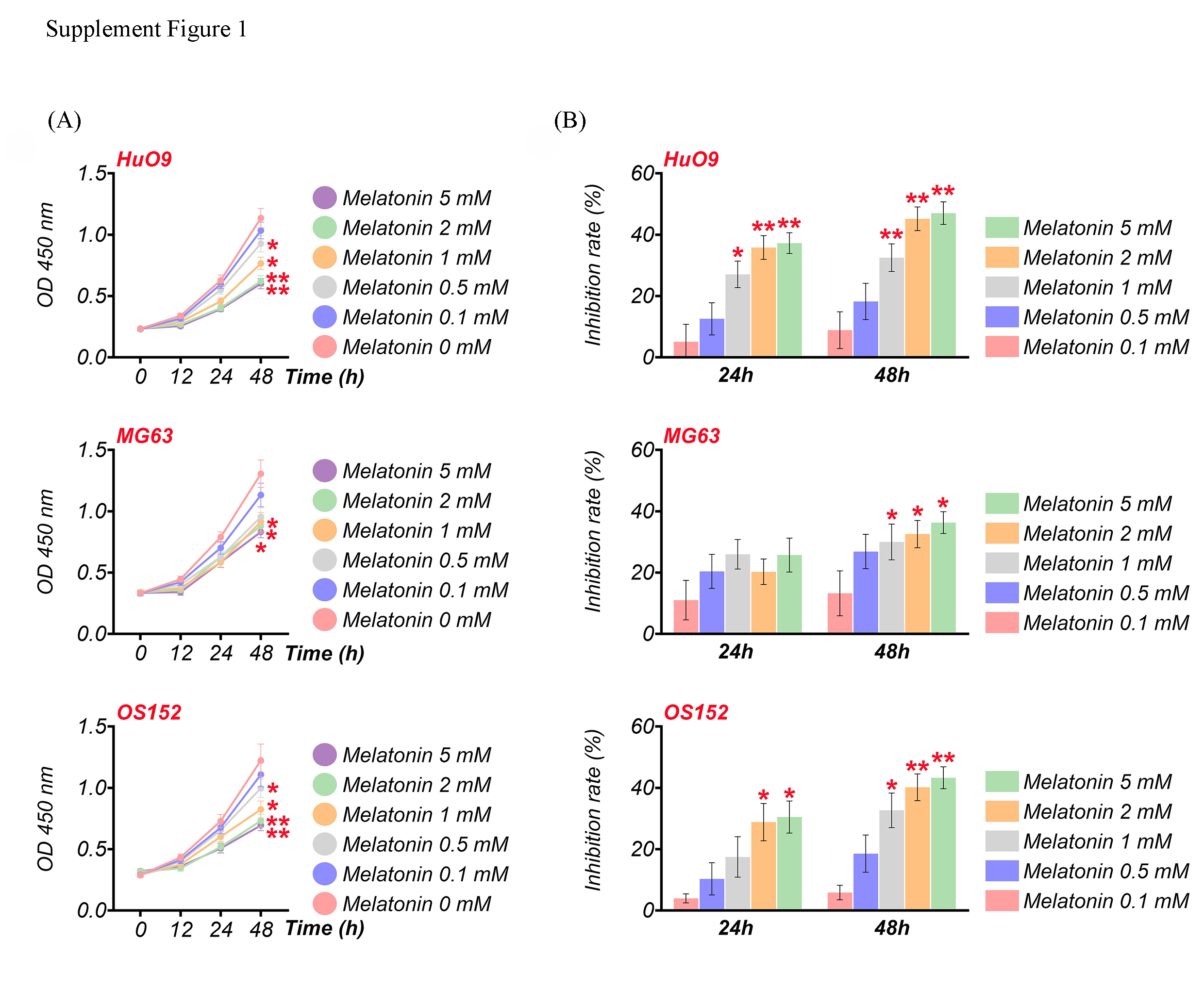

Supplement: Supplementary Figure 1 — Effects of melatonin on cell viability and proliferation inhibition in osteosarcoma cell lines. (A) Line graphs showing OD450 values of (top) HuO9, (middle) MG63, and (bottom) OS152 cell lines treated with increasing concentrations of melatonin at different time points. Data are presented as mean ± SD from three independent experiments. Asterisks indicate significant differences compared with the control group (0 mM melatonin) at the corresponding time point (*p < 0.05, **p < 0.01; two−way ANOVA followed by post hoc test). (B) Inhibition rates of HuO9, MG63, and OS152 cells after treatment with various concentrations of melatonin for 24 h and 48 h. The inhibition rate was calculated as [1 – (OD450 of treated group/OD450 of control group)] × 100%. Data are expressed as mean ± SD (n = 3). Significant differences relative to the control are marked with asterisks (*p < 0.05, **p < 0.01; multiple t−test or two−way ANOVA). [file Image1.tiff]
